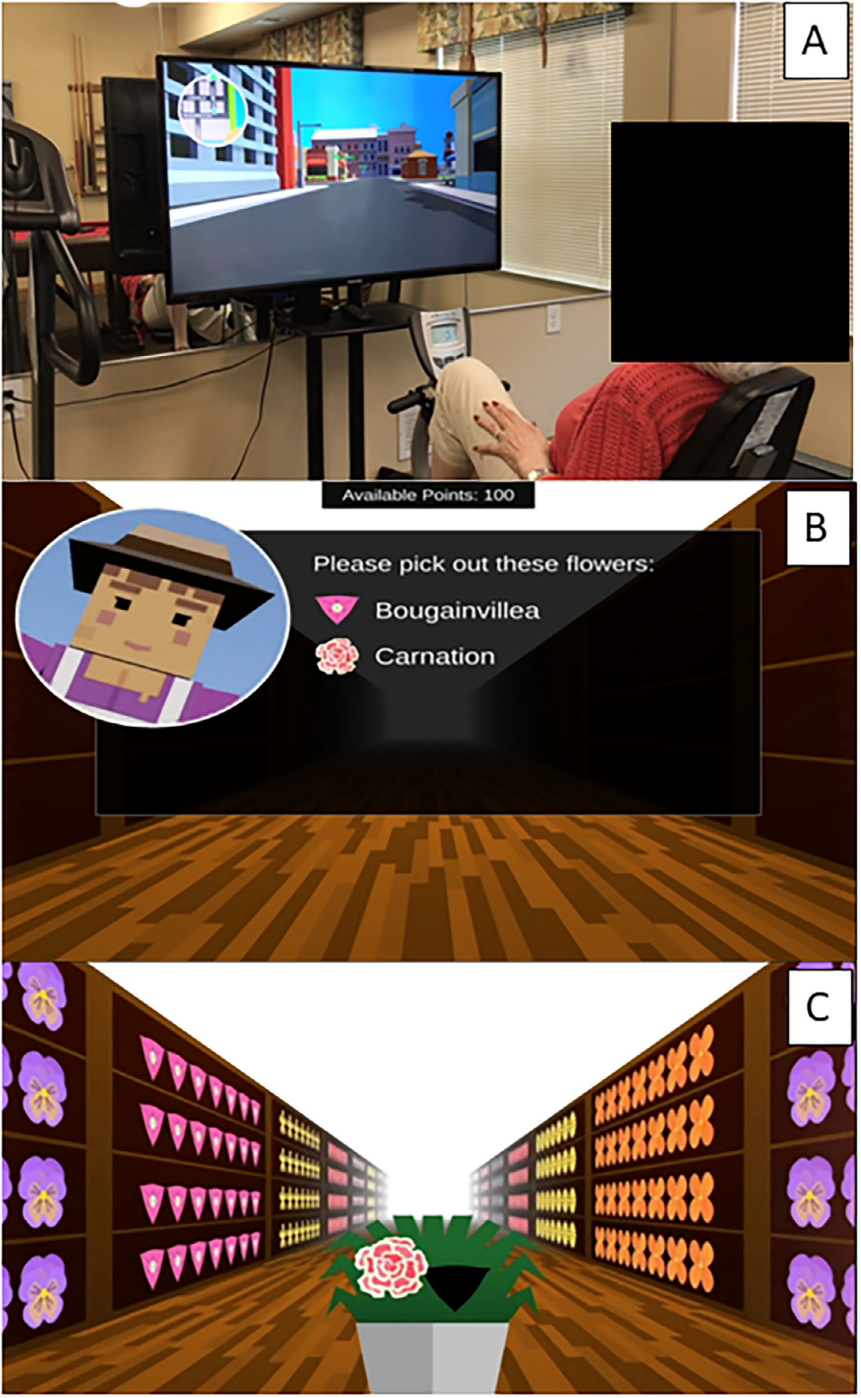# Preliminary Effectiveness of a Simultaneous, Aerobic Exercise and Cognitive Training Telerehabilitation Program in Subjective Cognitive Decline. The Exergames Study

**DOI:** 10.1002/alz.091541

**Published:** 2025-01-09

**Authors:** Dereck L. Salisbury, Keenan A Pituch, Fang Yu

**Affiliations:** ^1^ University of Minnesota, Minneapolis, MN USA; ^2^ Arizona State University, Phoenix, AZ USA

## Abstract

**Background:**

Subjective cognitive decline (SCD) represents an important therapeutic target to prevent future cognitive decline associated with aging as well as neurodegenerative diseases such as Alzheimer’s disease. One such therapy is the “dual‐task” exergaming with concurrent aerobic exercise (AEx) and cognitive training. The primary aim of this Stage IB randomized controlled trial (RCT) was to test the preliminary effects of a dual‐task exergaming telerehabilitation intervention on cognition and aerobic fitness, in comparison to AEx only and attention control (stretching) in older adults with SCD

**Method:**

This RCT randomized 39 participants on a 2:1:1 allocation ratio to supervised exergame (Figure 1) (n = 20), AEx (n = 11), and stretching (n = 8), 3 times a week for 12 weeks. The dual‐task exergaming was concurrent moderate‐intensity cycling and BrainFitRx® cognitive telerehabilitation. Cognition was assessed by NIH Toolbox Cognitive Battery and aerobic fitness by 6‐minute walk test (6MWT) and shuttle walk test.

**Result:**

The participants were 74.6 (7.4) years old with 17.7 (2.3) years of education, and 69% were female. The effect of time was significant, F(1,23.9) = 13.16, p = .001, for the Fluid Composite score, and significant within‐group changes were seen for the exergame group, t(14.08) = 2.53, p = .024, d = 0.33. Between‐group changes did not reach significant levels for any cognitive test. Between‐group changes for the 6MWT were not significant, but the change score (21m) seen in the exergame group is considered clinically significant change for older adults.

**Conclusion:**

The exergame participants further improved their fluid cognition, while the AEx and stretching groups did not, indicating a potential synergistic effect from AEx and cognitive training. The aerobic fitness changes were similar between the exergame and AEx only groups, indicating that the feasibility of adding cognitive training to AEx concurrently without sacrificing gains in aerobic fitness from AEx. Studies with larger sample sizes than our study are needed to duplicate our findings.